# Quest for Optimal Regression Models in SARS-CoV-2 Wastewater Based Epidemiology

**DOI:** 10.3390/ijerph182010778

**Published:** 2021-10-14

**Authors:** Parisa Aberi, Rezgar Arabzadeh, Heribert Insam, Rudolf Markt, Markus Mayr, Norbert Kreuzinger, Wolfgang Rauch

**Affiliations:** 1 Department of Infrastructure, University Innsbruck, 6020 Innsbruck, Austria; parisa.aberi@uibk.ac.at (P.A.); rezgararabzadeh@ut.ac.ir (R.A.); 2 Department of Microbiology, University Innsbruck, 6020 Innsbruck, Austria; Heribert.Insam@uibk.ac.at (H.I.); Rudolf.Markt@uibk.ac.at (R.M.); Markus.Mayr@uibk.ac.at (M.M.); 3 Institute for Water Quality and Resource Management, Technology University Vienna, 1040 Vienna, Austria; norbkreu@iwag.tuwien.ac.at

**Keywords:** regression, SARS-CoV-2, wastewater-based epidemiology, incidence, multivariate model, Taylor diagram

## Abstract

Wastewater-based epidemiology is a recognised source of information for pandemic management. In this study, we investigated the correlation between a SARS-CoV-2 signal derived from wastewater sampling and COVID-19 incidence values monitored by means of individual testing programs. The dataset used in the study is composed of timelines (duration approx. five months) of both signals at four wastewater treatment plants across Austria, two of which drain large communities and the other two drain smaller communities. Eight regression models were investigated to predict the viral incidence under varying data inputs and pre-processing methods. It was found that population-based normalisation and smoothing as a pre-processing of the viral load data significantly influence the fitness of the regression models. Moreover, the time latency lag between the wastewater data and the incidence derived from the testing program was found to vary between 2 and 7 days depending on the time period and site. It was found to be necessary to take such a time lag into account by means of multivariate modelling to boost the performance of the regression. Comparing the models, no outstanding one could be identified as all investigated models are revealing a sufficient correlation for the task. The pre-processing of data and a multivariate model formulation is more important than the model structure.

## 1. Introduction

For decades, data collected from the inflow of wastewater treatment plants (WWTPs) have been recognised as an important source of information for the detection of human diseases and/or drug abuse [1,2,3,4]. Consequently, for the Sars-Cov-2 pandemic as well, multiple studies have found wastewater-based epidemiology (WBE) as a potential tool [5,6,7,8,9,10] for the monitoring and management of the disease. The virus signal found in wastewater is the true prevalence information, that is, information on all infected persons in the watershed [11,12,13,14]. However, official reporting and statistics relies on the data derived from individual test programs, which includes only a subset of the overall infection and is a function of the test strategy. The difference to the true number of infections (as determined by prevalence data) is significant due to the high number of asymptomatic and mildly infected patients [15]. Still, the latter information serves as the backbone of SARS-CoV-2 management and is usually denoted as the incidence value, typically given as the 7- or 14-day notification rate of new infections for 100,000 inhabitants.

Despite the differences in the properties of the data (WBE prevalence and test-based incidence), studies have reported a significant statistical agreement between the two [16,17], thus indicating the capability of wastewater data for the prediction of COVID-19 incidence data [18]. In terms of pandemic management, this is a profound advantage both as supplement and alternative to individual testing. Furthermore, WBE was found to give a slightly earlier signal as compared to the clinical recognition of SARS-CoV-2 [19,20,21]. Thus, a mathematical model capable to predict the incidence values from the wastewater signal is a valuable tool in pandemic management.

Whereas a simple statistical model, such as a linear regression, can correlate two signals, a thorough analysis of the mechanisms and assumptions of the models are key for a robust prediction [22,23]. For instance, in addition to the observed latency between two signals, it was shown that the inclusion of metadata on the amount of testing might have a positive effect on the model performance. It is evident that increasing the number of tests results in the identification of a higher number of positive cases. However, owing to a lack of comprehensive community monitoring [24,25,26]; untested asymptomatic individuals, who comprise a significant number of patients [27,28,29,30,31]; and data security concerns, detailed test data—allowing for prevalence estimation—is unavailable on a general basis, thereby restricting the development of rigorous statistical models.

In relation to WBE modelling, researchers have taken advantage of regression models as a powerful technique for predicting and identifying the use and spread of substances in the population [32,33]. Regarding COVID-19, [10,34,35,36,37,38,39] published statistical models for the prediction of the number of active cases based on clinical information. However, only a few studies were dedicated to the regression between the viral load from wastewater and incidence values from individual testing [40,41]. The time latency between two signals, when considering the testing data, and the role of appropriate signal treatments such as signal filtering [42] and population-based normalisation [43,44] have typically been disregarded.

In this study, we go beyond a simple application of regression but aim to find optimal machine learning techniques to predict COVID-19 incidence. The innovation of the paper is the development and application of the rigorous methodology to derive the optimal model structure. We used monitoring data from four prototypical wastewater treatment plants in Austria to achieve the task.

## 2. Methodology

### 2.1. Modelling Procedure

**Figure 1:**
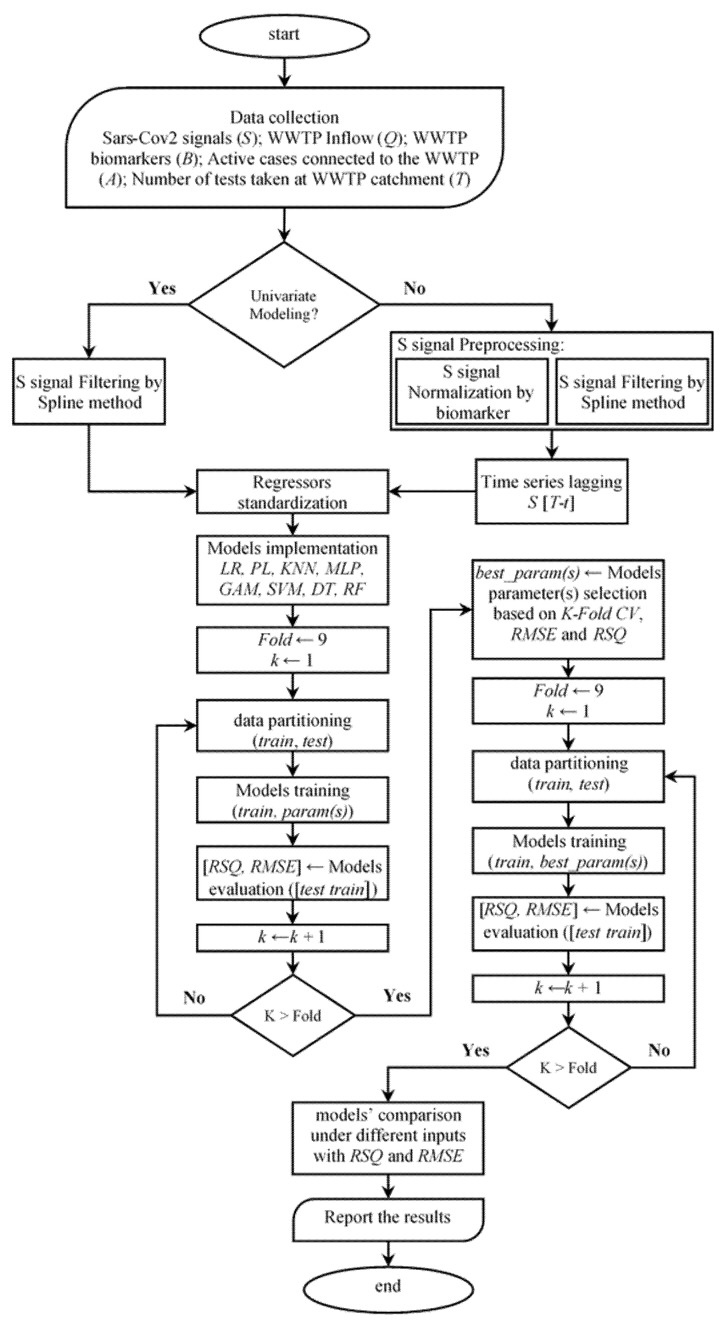
Modelling workflow.

To implement and analyse the various regression models, a standardised modelling procedure was applied, as depicted in Figure 1. The investigation follows two categories depending on the data availability and pre-processing.

- In the first category of the proposed modelling algorithm, the only source of information is the timeline of the wastewater signal, that is, the SARS-CoV-2 N1-gene copy numbers. Typically, this signal comes with significant noise and outliers, which influence the model training performance [45] Thus, a time series filtering method (the Spline method is used herein; [46]) is applied to filter out the noisy information adhered to the gene copy numbers.
- In the second category, in addition to the gene copy number, the information on the number of tests taken in the communities is used where available (in only two of the case studies). In addition, we apply further data pre-processing steps such as population-normalisation of the viral load, time lag, and time series filtering (as in the first category), the details of which are discussed in Section 2.3, Section 2.4 and Section 2.5.

For both categories, the regressors were standardised because statistical models are sensitive to the data scales and units [47].

Parametric statistical models must be provided with basic parameter information prior to training, for example, the number of neighbours in the K-nearest neighbour method (KNN). To tune these parameters, a range of feasible values was initiated for each method investigated. Next, for each (parameter) option, a *k*-fold cross validation [48] is performed to assess the model performance, where the regressors are shuffled and split into training/testing subsets. Given the selected statistical models with different structural architectures, the models are trained and tested for both calibration and validation horizons. The regressors are then re-shuffled and partitioned for *k* trials, and the models are recalibrated and evaluated for each trial. Based on the model performance for all trials (folds) of the test subset, the optimum parameter values are determined according to the performance indicators (PI), that is, the mean and variance of the root mean square error (RMSE) and the Pearson’s correlation coefficient (R^2^).

Given the calibrated parameter values, for example, the number of neurons for an ANN-MLP, a *k*-fold cross validation is conducted again to estimate the model performance metrics under different subsets of data to compare model performances.

### 2.2. Dataset

The data used in this study stem from two different sources, that is, health agency information and wastewater-based epidemiology measurements taken at four treatment plants across Austria. The information taken from health services includes the daily time series of the number of COVID tests (total and positive cases) as well as the number of deaths and recovered patients. The number of currently active cases (*A*) is computed by the sum of daily confirmed cases ($N_{c}$), the sum of deaths ($N_{d}$), and the sum of (infected and then) recovered patients ($N_{r}$) over the whole data series as follows:

(1) $$A=\sum N_{c}-\sum N_{d}-\sum N_{r}$$

**Figure 2:**
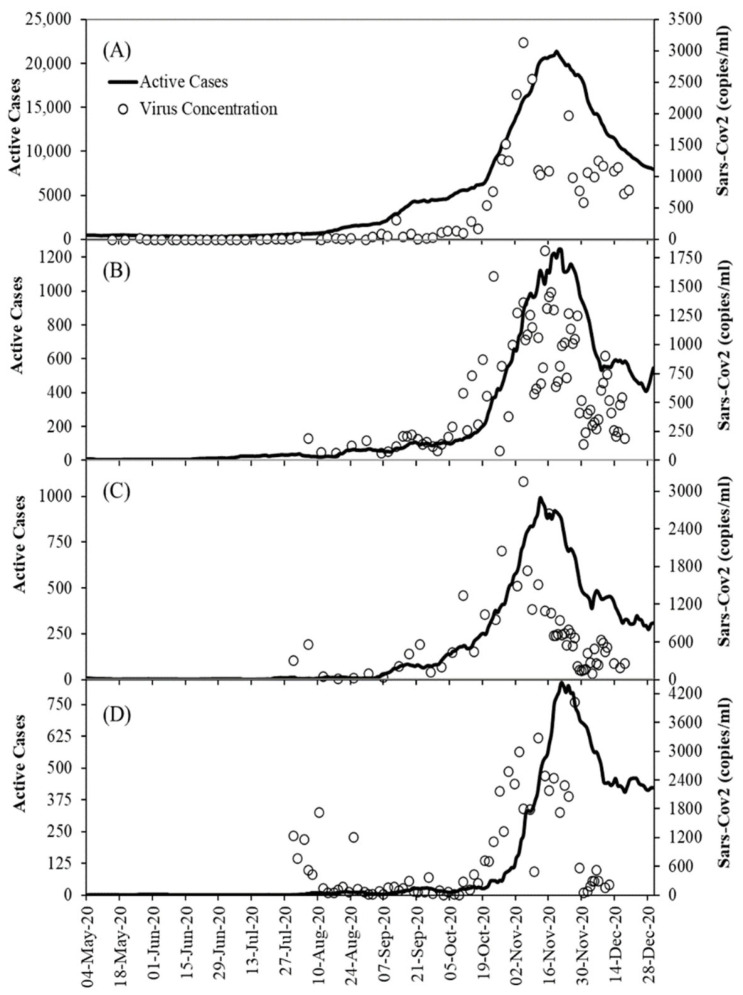
Raw data timeline of SARS-CoV-2 gene copy numbers (copies/mL) and epidemiological timelines at the four sampling sites, where (**A**–**D**) corresponds to WWTPs A–D.

While incidence is typically expressed as the sum of new infections over a period of 7 or 14 days and scaled to 100,000 inhabitants, we use in the following “active cases” (*A*) as proxy. The advantage is that parameter *A* accounts directly for the duration of the infection and not by arbitrary summation over 7 or 14 days. From the treatment plants, not only are SARS-CoV-2 signals available, so are the inflow data and NH_4_-N concentration, which are used as population markers. The WWTPs are anonymised by the letters A–D, with the mean of the data given in Table 1 and the timeline of the raw SARS-CoV-2 signal as well as the active cases depicted in Figure 2. Details of the measurement programs are given elsewhere [21,45] and not repeated herein. Note that the wastewater composition in these four case studies is in the range typically found in industrialised countries and will thus not influence the methodology.

Note that the information on health services (regarding the test regime and active infection cases) is regional and is thus not entirely accurate for the WWTP catchments in question. That is, whereas the information on the urban environment coincides with the drained catchment, which is less accurate for suburbs and scattered communities. For instance, the calculated active cases for WWTP ‘*B*’ (Table 1) belong to the urban sub-catchment of the plant coverage area but only approximately. Half of the total number of active cases were measured at WWTP ‘*B*’. However, assuming $B_{t}$ and $b_{t}$, as the time series of the number of active cases corresponding to the WWTP network and its main community, respectively, it was found that there is a nearly perfect linear relationship between $B_{t}$ and $b_{t}$ with a constant multiplier $B_{t}=k*b_{t}$. For the WWTPs, ‘*A*’, ‘*C*’, and ‘*D*’, the multiplier was calculated as 1≤k<1.2, and for ‘B’, we derive k=2. Given the linear relation, we must obtain a similar performance of the regression models for using a subset of the health information data instead of the accurate information.

### 2.3. Normalisation

Wastewater epidemiology assumes that each infected person sheds a certain amount of virus into the sewer network. For the normalisation of the SARS-Cov-2 signal, we compute the (theoretical) daily virus load per person based on the virus measurements in the WWTP by neglecting virus loss in transport and sampling:

(2) $$L_{virus}=\frac{c_{virus}*Q_{t}}{P_{t}}$$

where $L_{virus}$ is the daily virus load per person [genomes/cap/d], $c_{virus}$ is the measured virus concentration at WWTP [genomes/m^3^/d], *Q* is the inflow to the WWTP [m^3^/d], and *P* is the population connected to the treatment plant. To estimate the dynamics of the population in the catchment, population-based biomarkers are suggested [43,44]. To capture the temporal dynamics, we assume that the population can be estimated by means of the daily personal biomarker load $f_{bm}$ [M/m^3^/d/cap] as follows:

(3) $$P_{t}=\frac{c^{bm}*Q_{t}}{f_{bm}}$$

where $c^{bm}$ is the biomarker concentration (M/m^3^). Substituting Equation (3) into Equation (2) gives

(4) $$L_{virus}=f_{bm}\frac{\;c_{virus}}{c^{bm}}$$

The use of an appropriate biomarker is the subject of many studies [49]; however, in this study, NH_4_-N was routinely monitored. To estimate the representative value $f_{NH4N}$, the 50th percentile of the daily personal NH_4_-N load was determined during the period of the first lockdown in Austria (16 February to 13 April 2020) because the inflow fluctuations to the WWTPs are minimal during that period (see also [45]). *f_NH4N_* was found to range between 6.5 and 10.7 g NH4_N/m^3^/cap/d.

### 2.4. Filtering

The time series of both raw and normalised surveillance data comes with stochastic noise, resulting in misleading outputs caused by an overfitting to noisy data. In this study, the Spline algorithm was used to filter both the raw and normalised SARS-CoV-2 signals. The applicability and details of the Spline technique are widely discussed [50,51] and are also suggested by [45] specifically for WBE. The parameters for Spline smoothing are determined by calibration, using performance indicators such as mean absolute error, variability and Akaike information criterion [45].

### 2.5. Lagging

There is a temporal mismatch between the virus load measured at WWTP and the virological information released from clinical studies [21] which lags behind the wastewater signal. To adjust the time difference between the measured signals at the WWTPs and confirmed (active) cases, a cross-correlation analysis (CCA) was conducted to determine the best lagged explanatory variables. CCA is applied to a range of lagged time-series to determine significantly correlated signals with a at the level of 95% confidence. The CC significance limit is calculated as below [52]:

(5) $$l=\frac{\pm 1.96}{\sqrt{n}}$$

where *n* is the number of observations. With the derived candidate lags (approximately 2–10 days) from the CCA, a linear model (LM) and ANOVA was set up for all possible combinations of lagged variables and the number of clinical tests where available. Finally, among the statistically meaningful model structures, we selected those with the lowest Akaike information criterion [53].

### 2.6. Regression Models

In this study, eight regression models that are popular in science and engineering prediction problems were evaluated and compared to predict their capability. The structure of the models in other studies and their application to COVID-19 modelling are presented in Table 2.

### 2.7. Evaluation

Under data limitations, a cross-validation procedure is applied to evaluate the performance of the regression models [67]. With this method, data are randomly shuffled and divided into training/testing subsets for model calibration and assessment. This is iterated *k*-times (*k*-fold) to ensure that the sampling process is unbiased. Herein, we apply a nine-fold cross-validation scheme to assess each implemented model. The results of a k-fold cross-validation are summarised with the mean and variance of the squared Pearson’s correlation coefficient (R^2^) and root mean square error (RMSE). The equations for R^2^ and RMSE are presented in the Appendix A.

## 3. Results and discussion

### 3.1. Time Series Lag

**Figure 3:**
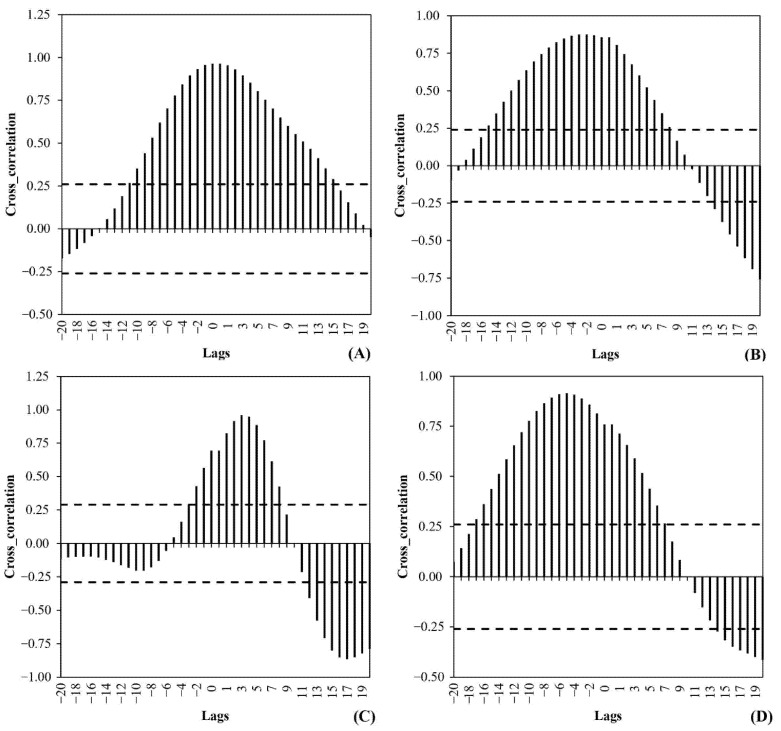
Cross-correlation (CC) plots between SARS-CoV-2 signals and active cases in WWTPs (**A**–**D**). The negative and positive lags correspond to the forward and backward lags between the incidence time series and viral load time series. The solid dashed lines indicate the significant levels at 95% confidence.

To identify the existing time lags between the viral load and the clinical information (see Section 2.5), the cross-correlation shown in Figure 3. Using Equation (5), CC significance limits are calculated, varying from ±0.25 to ±0.04.

For instance, in WWTP A, there is a significant correlation of between −11 and +15 days, whereas in WWTP B, it is approximately −15 to +8 days. Similarly, in WWTP D, a range of −16 to +7 days is computed as significant time shifts, whereas WWTP C has a significant time shift of −3 to +8 days. In general, in WWTPs A, B, and D, the corresponding CC plots show a similar time shift of (−14 ± 3) to (+10 ± 5), indicating WWTP C with different time-series characteristics.

In compliance with the objective of this study, which is the establishment of predictive regression models for active cases based on WWTP data, the forward correlation lags are neglected and model regressors are populated based on active backward-lagging time-series cases. To this end, assuming a bivariate model input, the combinations of active time-series cases with different lags were considered and analysed using an analysis of variance (ANOVA). For each treatment plant and under a confidence of 95%, the best time-series lags were selected using AIC [53]. In addition to the viral load, the number of tested persons available in the catchment of two WWTPs, A and B, is also considered as an ANOVA input.

Table 3 presents the results of the best linear model structures derived using AIC. Based on the table, the adjusted R2 values for WWTPs A and B are approximately 8% higher than those for WWTPs C and D, denoting the importance of the additional exogenous variable (number of tests), information that is missing for WWTPs C and D. Under a 95% confidence level, the model parameters (with the exception of the model A intercept) contribute significantly to the explanation of their respective variance in active cases. According to the results, SARS-CoV-2 signals with lags of (2, 4), (2, 7), (5, 7), and (3, 7) were found to be optimum regressors for the investigated cases.

**Figure 4:**
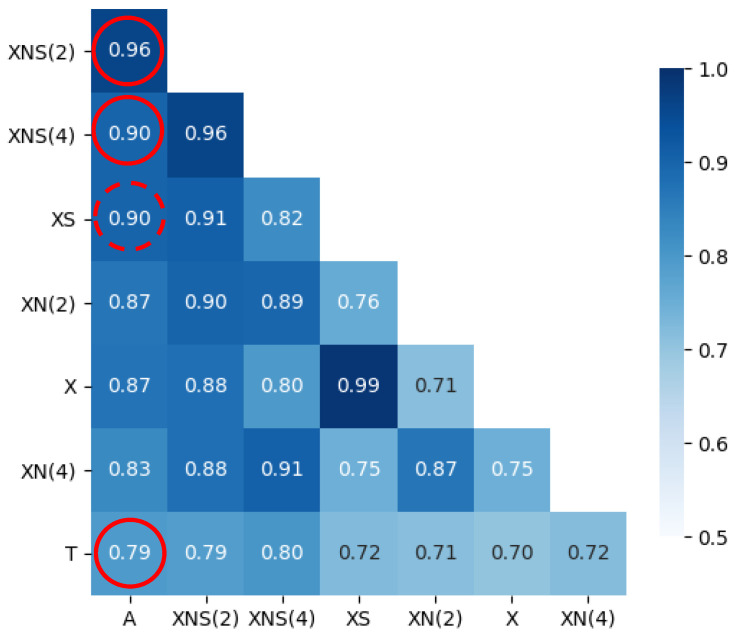
Correlation matrix for model regressors. Solid circles indicate Pearson’s correlation coefficients for multivariate models, and the dashed circle shows the same metric for a univariate model. Notations: A, active cases; X, SARS-CoV-2 load; N, normalised signal; S, smoothed signal; T, number of tests taken; and (number), a signal with number time step delay.

The heat map shown in Figure 4 allows the identification of the significant values of the Pearson correlation coefficient [68] for different input signals: univariate (category 1) and multivariate (category 2) models.

### 3.2. Global Parameter Tuning

**Figure 5:**
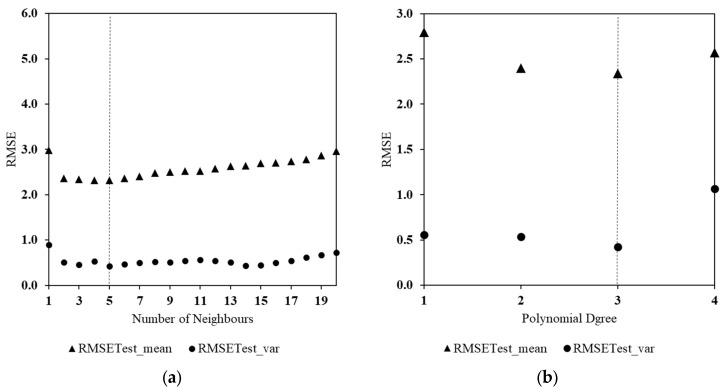

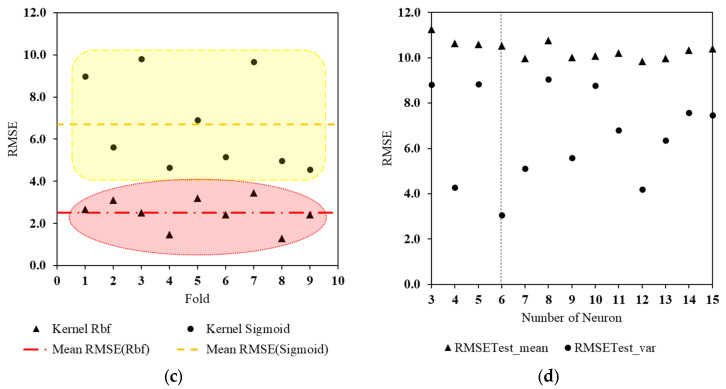
Evaluation of regression models ((**a**) KNN, (**b**) polynomial, (**c**) SVR, (**d**) MLP), for different ranges of parameters using the mean and variance of RMSE for the test data—WWTP A.

Given that most of the selected statistical models are parametric, a grid search is conducted to find the basic setup values that are optimum for those parameters (e.g., number of neurons). Accordingly, a multitude of regression models for different parameter ranges were evaluated. The models were calibrated and evaluated for the test subsets based on the mean and variance of the RMSE performance indicator. Figure 5 shows the resulting metrics of nine-fold randomly selected test subsets against the calibrated setup parameters of the models (e.g., KNN, MLP, SVR, polynomial, decision tree, and random forest) applied to the WWTP A data:

For example, as indicated in Figure 5, the order of the polynomial method and the depth of the random forest are calculated as 3 and 2, respectively. The same process was applied to all models at all WWTPs to ensure the model optimality.

Regarding the actual values of the parameters a comparison (and analysis of variation) is not meaningful as the values differ widely—especially for complex models. Only for linear models could a more rigorous comparison be conducted, but also here the variations are heavily influenced by the time lag. To conclude, it is not possible to derive a unique set of parameter values that result in a fair overall regression, but tuning is necessary for each site.

### 3.3. Model Metrics

**Figure A1:**
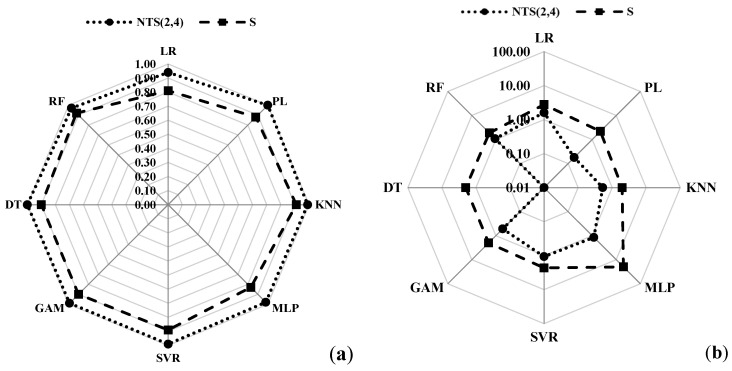
Model performance as spider chart—WWTP A. (**a**) RMSE, and (**b**) RSQ metric comparing signal (*S*) only with combined information of signal (S), normalisation (*N*) and tests taken (*T*).

Evaluating the regression models for the two input data categories (as described in Section 2.1), the R^2^ and RMSE metrics calculated for the different statistical models are plotted in Appendix B Figure A1 for the example of WWTP A. It is worth mentioning that the PI values for each station are based on the average of the metrics over a nine-fold trial. Models containing additional information (category 2) outweigh models that are fed only with a filtered SARS-CoV-2 signal (*S*), proving the importance of signal lagging, normalisation (*N*), and the number of tests taken (*T*) within the target communities. An increase in the model performance of approximately 10% to 20% and 1.0% to 5.0% in terms of R^2^ and RMSE, respectively, is observed (data found in Supplementary Materials Figures S1–S3).

**Figure 6:**
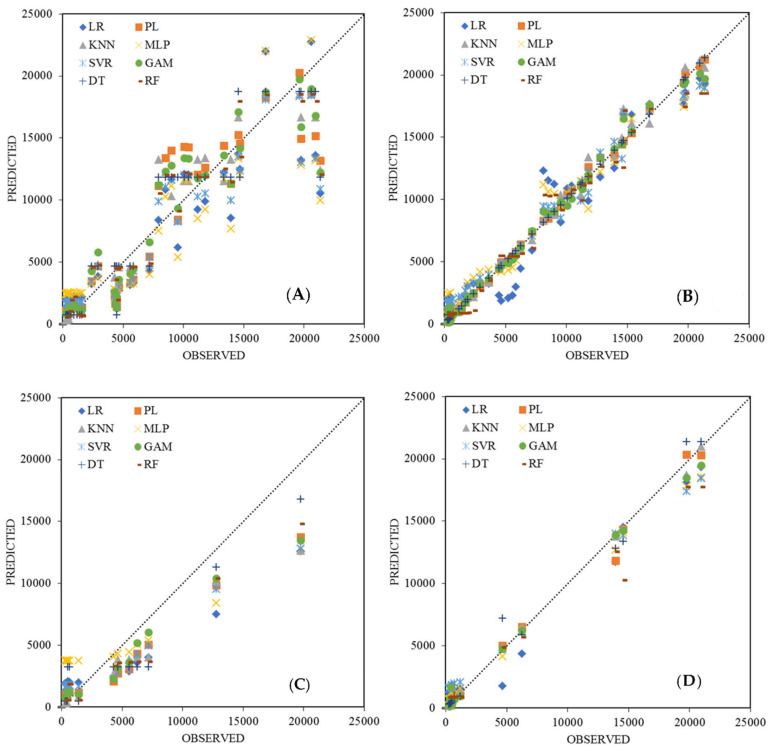
Visualisation of the number of active cases recorded versus model prediction in WWTP A: (**A**,**B**) model predictions against recorded data for training subset under univariate and multivariate inputs, respectively, and (**C**,**D**) the same plots for the testing subset.

Although the improvement varies for the different regression models, it can be deduced that all models perform better with additional information. In Figure 6, the model predictions regarding active cases are plotted for all implemented regression models in both univariate and multivariate approaches for the testing/training subsets for WWTP A. As shown in the figure, all models depict a superior fitness under the multivariate approach, particularly in terms of bias. An evaluation of the models for the test subset indicates a significant underestimation in the univariate models, whereas when applying additional information and conducting a multivariate analysis, a smaller bias is observed. Despite the improvement in the model metrics with multivariate data inputs, LR achieves the worst performance in both the training and test subsets. Because models such as RF, DT, and MLP tend to overfit in terms of calibration, they propagate larger biases during testing. Conversely, models such as KNN, PL, SVR, and GAM achieve an outstanding performance for both the testing and training subsets because they prevent an overfitting during the calibration. The results for the other three WWTPs are included in the Supplementary Materials (Figures S4–S6), the patterns of which are similar.

### 3.4. Model Comparison

**Figure 7:**
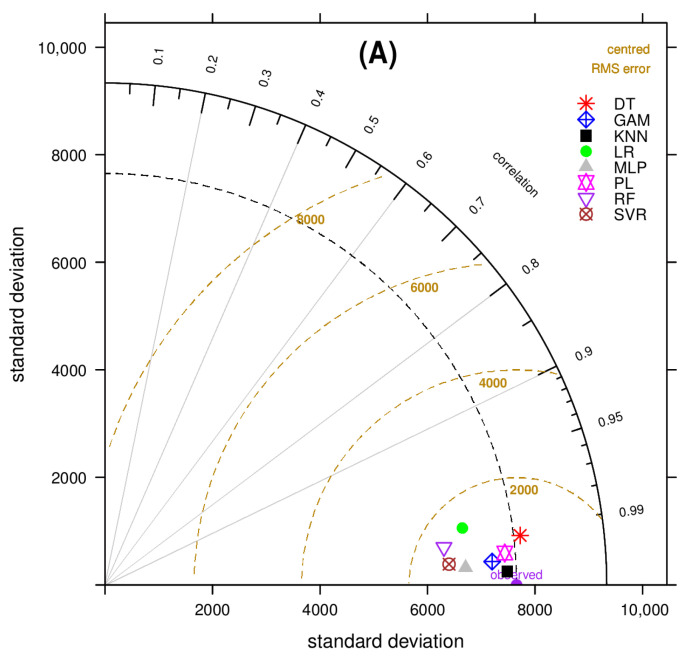
Taylor diagram, displaying statistical comparison of the eight model predictions against the actual number of recorded active cases—WWTP A.

To measure the degree of correspondence between the observed values and the predictions by the implemented models, a Taylor diagram (TD) [69] was drawn for the multivariate models under the test horizon (Figure 7). The closer the plotted model to the *x*-axis (indicating the observations), the lower the relative RMSE and the higher the correlation. The WWTP A, KNN, GAM, and PL models are closer to the point marked ‘observed’ data, with KNN appearing to be optimal. The TDs for the other three WWTPs are presented in the Supplementary Materials (Figure S7).

The TD of WWTP B indicates that the KNN, GAM, and PL models achieve a higher prediction accuracy, and KNN was again found to be the best option. For WWTPs C and D, the three best-simulated models are MLP, PL, and SVR, and SVR, KNN, and PL, respectively, where MLP and SVR are selected as the best regression models. In summary, Table 4 makes the following key conclusions from the Taylor diagram analysis: (i) the PL model is found most frequently to be among the top three options across all studied WWTPs, with a slight difference from the best model; (ii) the KNN model is the best model in the two larger communities (WWTPs A and B), followed by GAM; (iii) in the smaller communities, namely C and D, the SVR model tends to be more accurate, and was found to be the best and second best in WWTPs D and C, respectively, and iv) all models operate satisfactorily on a general level, even the multivariate LR model.

**Figure 8:**
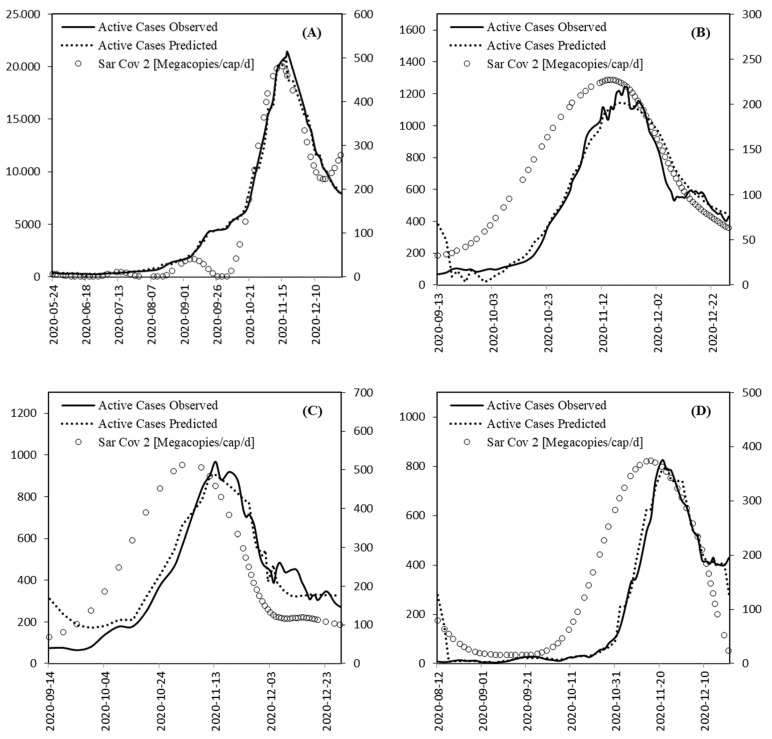
Data timeline of smoothed and normalized SARS-CoV-2 titer values (Megacopies/cap/d) versus active cases in observed/predicted timelines for the best models in WWTPs (**A**–**D**).

Based on the description in Section 3.3, and owing to the comparatively poor performance of the univariate models, only the results of the top-rated multivariate models categorised in Table 4 are shown in Figure 8. As can be seen, there is a better fit between the predicted values and the observed time series at WWTPs A and B, which is attributed to the availability of the number of tests taken in these case studies. Note that the timeline of the SARS-CoV-2 data in Figure 8 is the smoothed and normalized signal only, but without the influence of lagging—as taken into account in the modelling.

## 4. Conclusions

In the present study, we systematically investigated the correlation of the SARS-CoV-2 gene copy number found by WBE and the incidence values derived from individual testing. The study compared eight different regression models for a dataset monitored for four WWTPs in Austria. The key findings are as follows:

- There is a consistent time shift between the (earlier) wastewater signal and the clinical test records, varying from 2 to 7 days in our dataset—depending both on time period and site;
- A thorough pre-processing of the data, such as population-based normalisation and smoothing, leads to more robust models and is important for practical application;
- The inclusion of additional information (most importantly the time lag and number of tests taken) by applying multivariate models significantly increases the performance of all investigated models;
- All multivariate models are generally applicable for the regression, and even a simple linear regression can be used, despite showing the poorest performance.
- While the differences are small, PL and KNN outperform more complex models such as GAM, SVR, and MLP;
- As seen from above, regression between the wastewater signal and incidence values is derived easily—also, in a practical context. The information supplements—but could even replace—individual testing for incidence.

## Figures and Tables

**Table 1:** Summary of wastewater treatment plant datasets.

WWTP	Start Date	End Date	Avg. Daily SARS-CoV-2 Gene Copies/mL	Avg. Daily Active Cases	Avg. Daily Number of Tests	Avg. Daily Inflow (m^3^/d)	Population (1 January 2020)	Avg. Daily NH_4_-N (mg/L)
A	4 May 2020	30 Dec 2020	464	5325	4218	539,450	1,900,000	38.09
B	3 Aug 2020	28 Dec 2020	609	249	1126	83,187	320,681	29.49
C	27 Jul 2020	28 Dec 2020	658	186		16,344	41,696	28.90
D	27 Jul 2020	21 Dec 2020	781	136		4899	23,600	34.18

**Table 2:** Regression models applied to predicting the number of active cases.

Model	Reference	Application in Covid 19 Modeling
Linear (LR)	[54]	[34]
Polynomial (PL)	[55]	[56]
K Nearest Neighbor (KNN)	[57]	[58]
Multilayer Perceptron (MLP)	[59]	[36]
Support Vector Regression (SVR)	[60]	[61]
Generalized Additive Models (GAM)	[62]	[63]
Decision Tree (DT)	[64]	[65]
Random Forest (RF)	[15]	[66]

**Table 3:** Results of ANOVA for best linear model structures for WWTPs A–D.

WWTP	Models Metrics						
	R-Squared	Adj. R-Squared	F_Statistic	Prob (F-Statistic)	Log-Likelihood	AIC	BIC
A	0.939	0.936	303.600	0.000	−554.100	1116.000	1125.000
B	0.942	0.939	365.600	0.000	−425.690	859.400	868.500
C	0.868	0.862	148.300	0.000	−280.880	567.800	573.400
D	0.873	0.869	196.800	0.000	−356.860	719.700	726.000
	Models parameters						
	Const.	S2 *	S3	S4	S5	S7	T **
A	573.150	49.890	-	−16.710	-	-	0.003
B	−278.660	3.230	-	-	-	2.040	0.001
C	167.425	-	-	-	2.482	−1.060	-
D	−69.215	-	1.035	-	-	0.916	-
	Parameters significance: P > │t│						
A	0.193	0.000	-	0.001	-	-	0.010
B	0.000	0.000	-	-	-	0.000	0.000
C	0.000	-	-	-	0.000	0.000	-
D	0.001	-	0.000	-	-	0.000	-

* SN: Sars-Cov2 Signal with ‘N’ days delay ** Number of persons tested in the WWTP catchment.

**Table 4:** Top-three regression models for prediction in each WWTP.

Model/WWTP	A	B	C	D
DT				
GAM	○	○		
KNN	●	●		○
LR				
MLP			●	
PL	○	○	○	○
RF				
SVR			○	●

●: The best model; ○: The second-best models.

## Data Availability

Publicly available data used in this study can be found at https://covid19-dashboard.ages.at (accessed on 10 February 2021).

## Supplementary Materials

The following are available online at https://www.mdpi.com/article/10.3390/ijerph182010778/s1, Figure S1: Model performance as spider chart—WWTP B. Left: RMSE and right: RSQ metric comparing signal (*S*) only with combined information of signal (S), normalisation (*N*) and tests taken (*T*). Figure S2: Model performance as spider chart—WWTP C. Left: RMSE and right: RSQ metric comparing signal (*S*) only with combined information of signal (S), normalisation (*N*) and tests taken (*T*). Figure S3: Model performance as spider chart—WWTP D. Left: RMSE and right: RSQ metric comparing signal (*S*) only with combined information of signal (S), normalisation (*N*) and tests taken (*T*). Figure S4: Visualisation of the number of active cases recorded versus model prediction in WWTP B: (i) and (ii) model predictions against recorded data for training subset under univariate and multivariate inputs, respectively, and (iii) and (iv) the same plots for the testing subset. Figure S5: Visualisation of the number of active cases recorded versus model prediction in WWTP C: (i) and (ii) model predictions against recorded data for training subset under univariate and multivariate inputs, respectively, and (iii) and (iv) the same plots for the testing subset. Figure S6: Visualisation of the number of active cases recorded versus model prediction in WWTP D: (i) and (ii) model predictions against recorded data for training subset under univariate and multivariate inputs, respectively, and (iii) and (iv) the same plots for the testing subset. Figure S7: Taylor diagram, displaying statistical comparison of the eight model predictions against the actual number of recorded active cases—WWTP B, C and D.

## Appendix A

The equation of the coefficient of determination is:

(A1) $$R^{2}=\frac{{\mathrm{SS}}_{\mathrm{res}}}{{\mathrm{SS}}_{\mathrm{tot}}}$$

where {\displaystyle R^{2}=1-{SS_{\rm {res}}\over SS_{\rm {tot}}}\,}$SS_{res}$ refers to the residual sum of squares and $SS_{tot}$ is the total sum of squares. In the ideal condition of any model, when both observation and simulated dataset are perfectly fitted, the result of R^2^ is 1.

To measure errors as a result of model prediction/fitness the RMSE is calculated as:

(A2) $$\mathrm{RMSE}=\sqrt{\frac{\sum_{i=1}^{N}{\left(X_{i}-{\hat{X}}_{i}\right)}^{2}}{N}}$$

where $X_{i}$ is the measured output, ${\hat{X}}_{i}$ is the model fitness/prediction output, and N is the number of observations. The lower RMSE, the better the fitness; becoming zero when the model fitness/prediction is perfectly matching the observation records.
