# Supplementary material for: Quest for Optimal Regression Models in SARS-CoV-2 Wastewater Based Epidemiology
Source: Int J Environ Res Public Health. 2021 Oct 14;18(20):10778. doi: 10.3390/ijerph182010778 (PMC8535556; doi:10.3390/ijerph182010778)
Supplement: Supplementary file 1 [file ijerph-18-10778-s001.zip › Supplementary Material.pdf]

International Journal of
Environmental Research and Public Health

MDPI

Supplementary material

Quest for optimal regression models in SARS-CoV-2 wastewater based epidemiology

Parisa Aberi[1], Rezgar Arabzadeh[1], Heribert Insam[2], Rudolf Markt[2], Markus Mayr[2], Norbert Kreuzinger[3] and Wolfgang Rauch[1*]

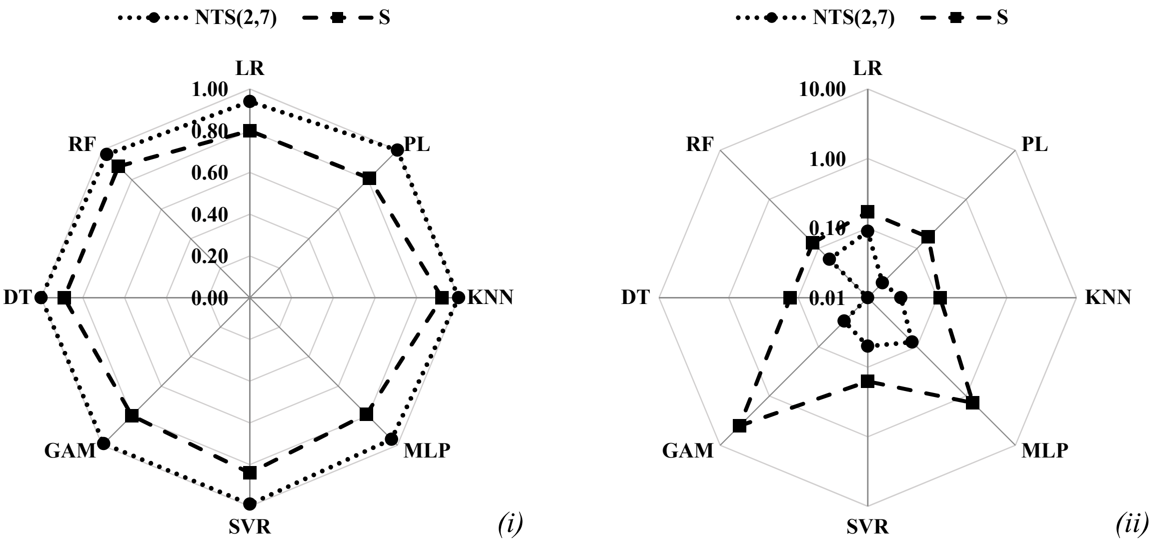

Figure S1: Model performance as spider chart – WWTP B. Left: RMSE and right: RSQ metric comparing signal (*S*) only with combined information of signal (S), normalisation (*N*) and tests taken (*T*).

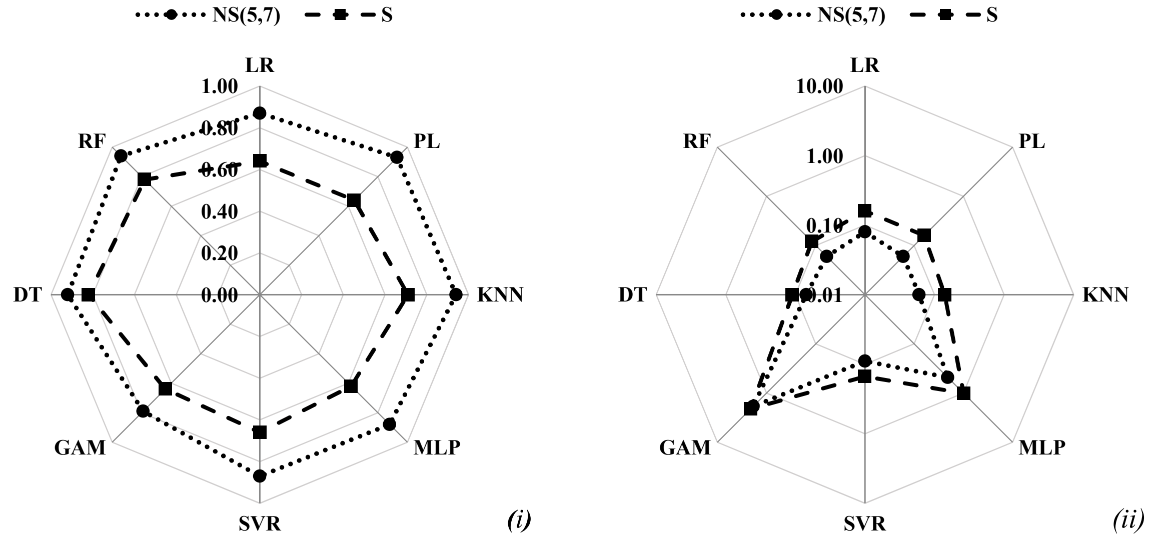

Figure S2: Model performance as spider chart – WWTP C. Left: RMSE and right: RSQ metric comparing signal (*S*) only with combined information of signal (S), normalisation (*N*) and tests taken (*T*).

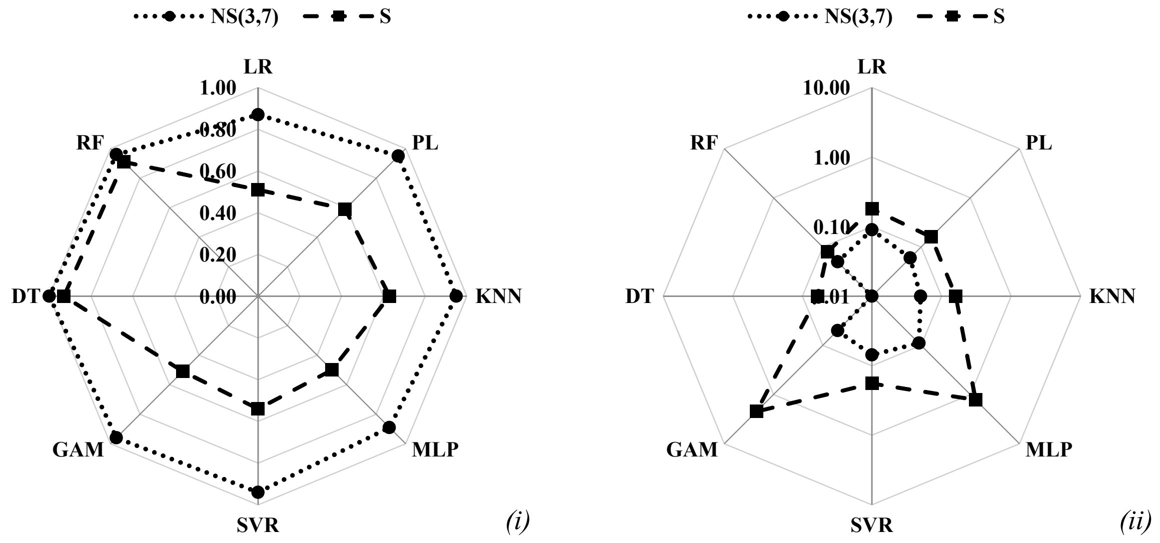

Figure S31: Model performance as spider chart – WWTP D. Left: RMSE and right: RSQ metric comparing signal (*S*) only with combined information of signal (S), normalisation (*N*) and tests taken (*T*).

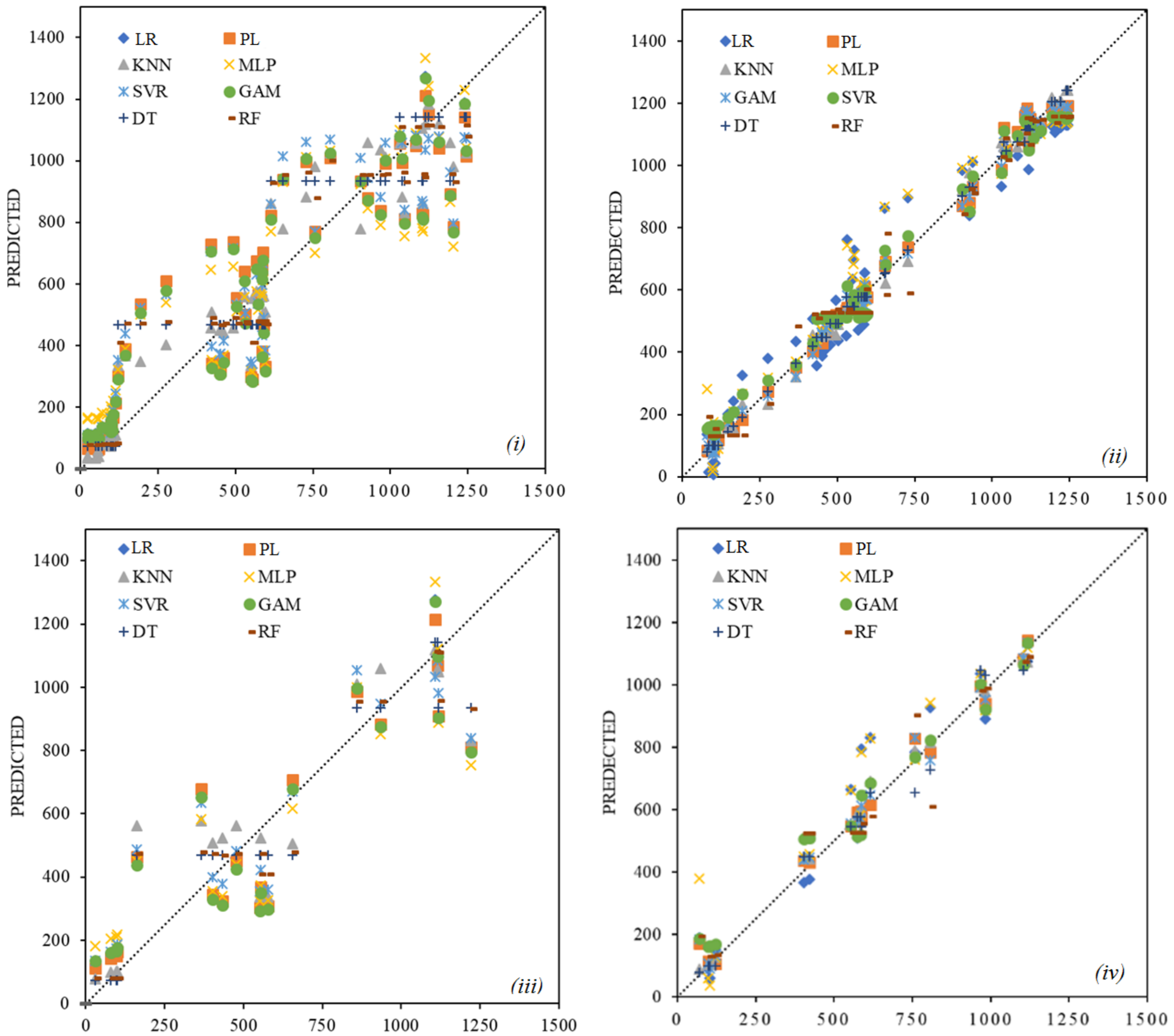

Figure S4: Visualisation of the number of active cases recorded versus model prediction in WWTP B: **(i)** and **(ii)** model predictions against recorded data for training subset under univariate and multivariate inputs, respectively, and **(iii)** and **(iv)** the same plots for the testing subset

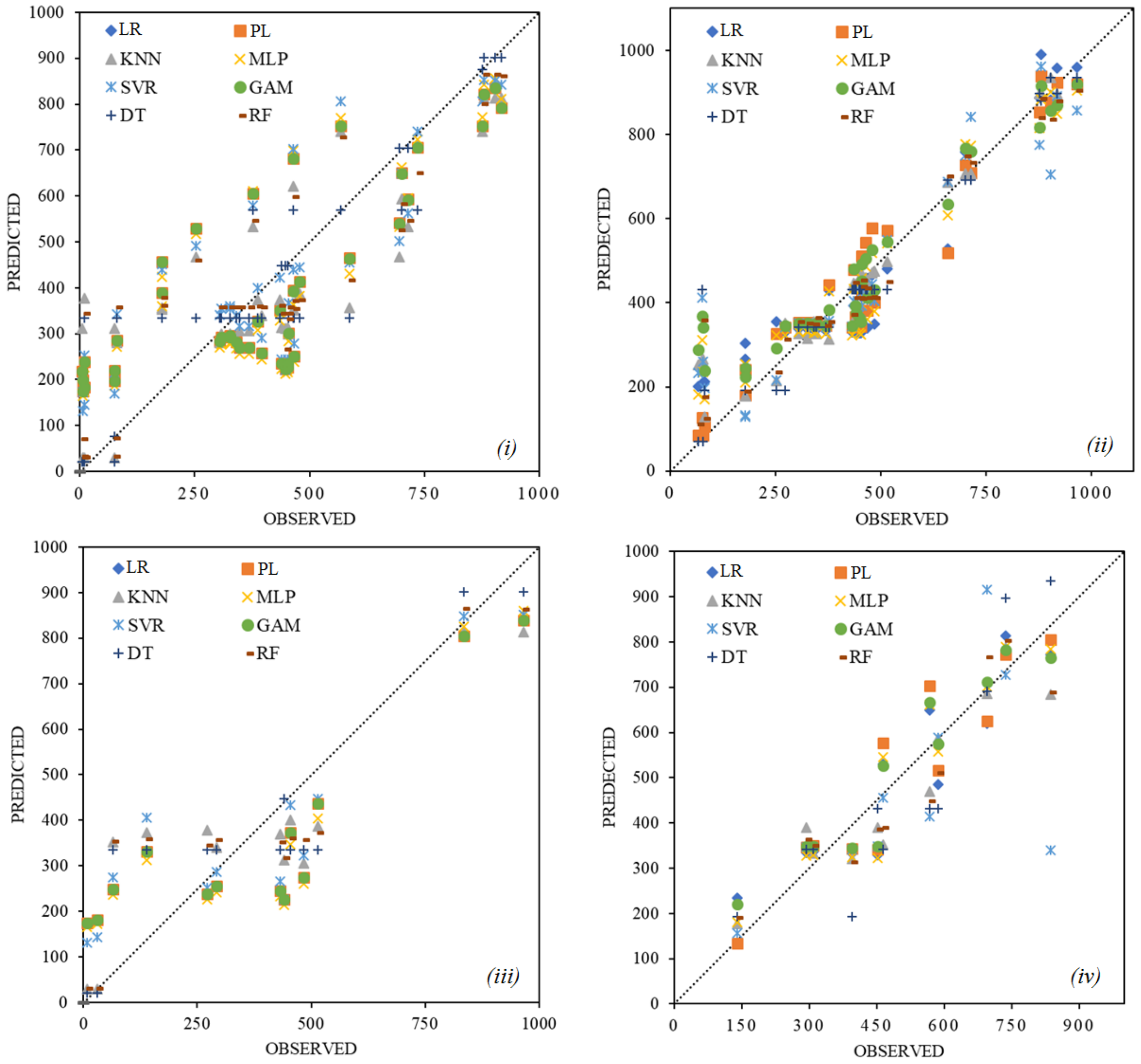

Figure S5: Visualisation of the number of active cases recorded versus model prediction in WWTP C: **(i)** and **(ii)** model predictions against recorded data for training subset under univariate and multivariate inputs, respectively, and **(iii)** and **(iv)** the same plots for the testing subset

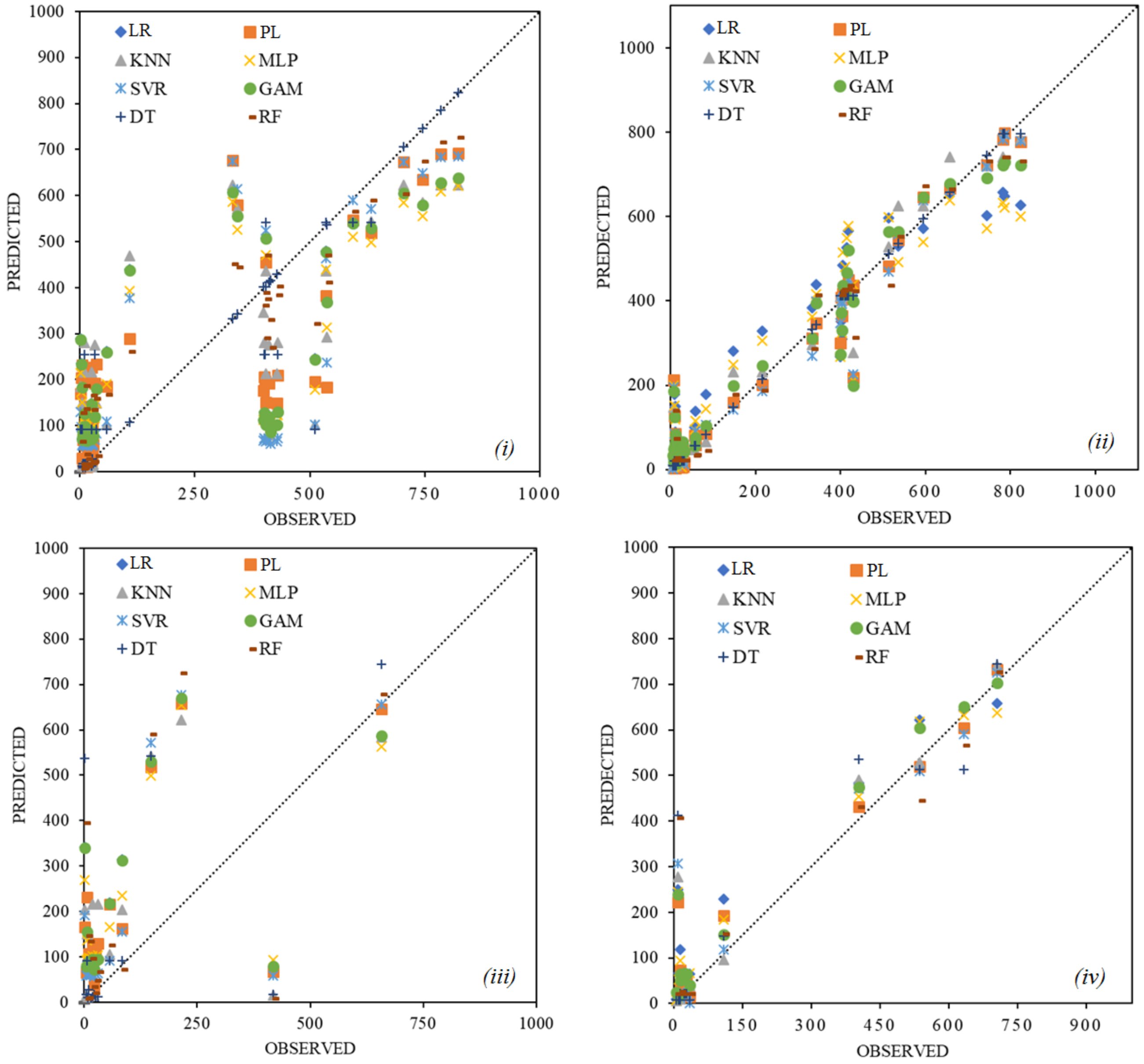

Figure S6: Visualisation of the number of active cases recorded versus model prediction in WWTP D: **(i)** and **(ii)** model predictions against recorded data for training subset under univariate and multivariate inputs, respectively, and **(iii)** and **(iv)** the same plots for the testing subset

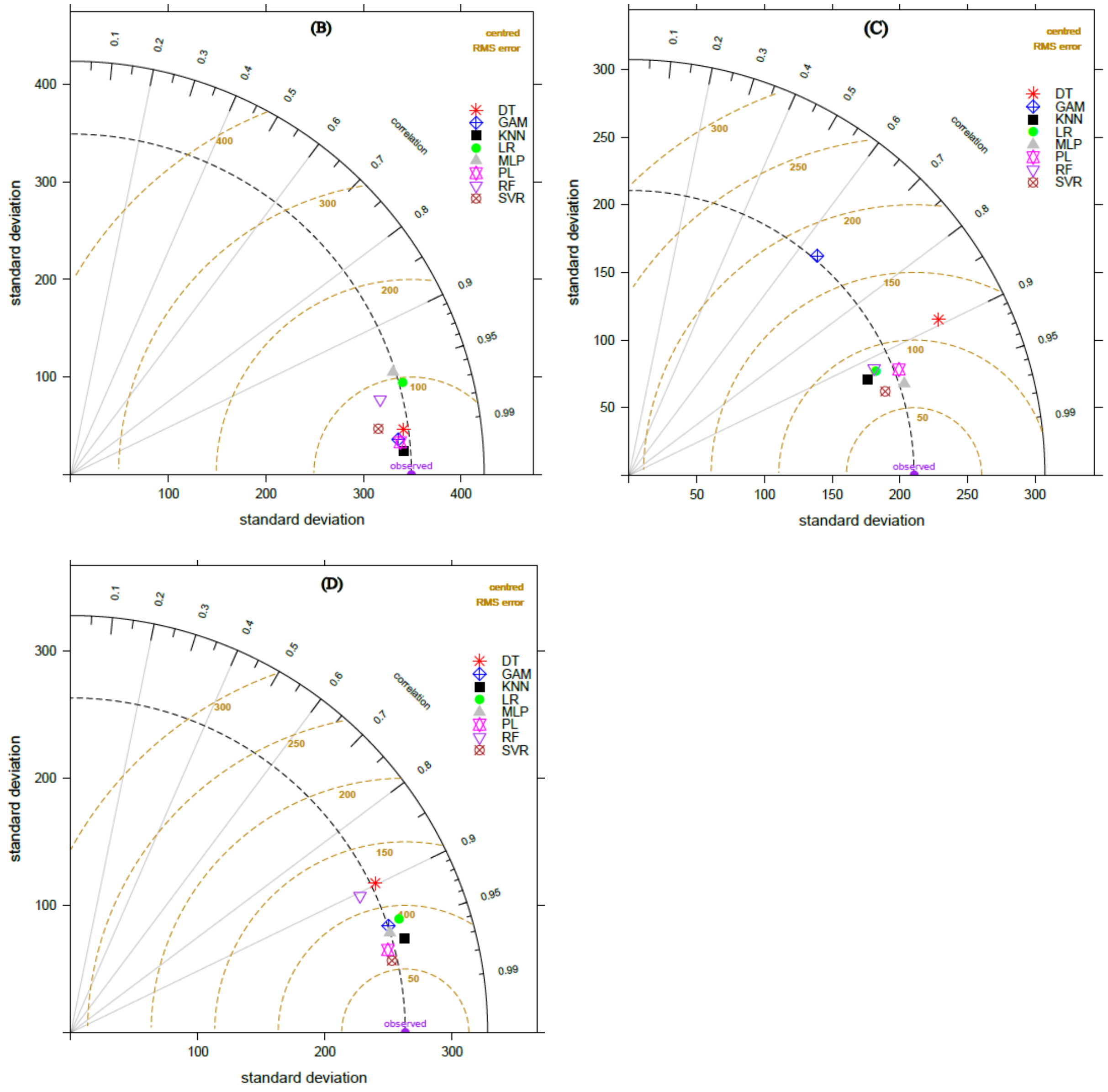

Figure S7: Taylor diagram, displaying statistical comparison of the eight model predictions against the actual number of recorded active cases - WWTP B,C and D
